# An intrapartum cervical buttonhole tear: A case report and review of rare tear pathogenesis

**DOI:** 10.1016/j.crwh.2023.e00516

**Published:** 2023-05-23

**Authors:** Amy Newnham-Hill, Joshua Odendaal, Catherine Hillman

**Affiliations:** Worcestershire Acute Hospitals NHS, Charles Hastings Way, Worcester WR5 1DD, United Kingdom

**Keywords:** Cervical tear, Postpartum haemorrhage, Surgical technique, Pregnancy, Instrumental delivery

## Abstract

Genital tract trauma and obstetric anal sphincter injuries are known complications of normal vaginal and assisted vaginal delivery. Cervical tears are an uncommon complication that can lead to significant postpartum haemorrhage and may have implications for future pregnancies. Careful evaluation of the genital tract, including the cervix, along with adequate resuscitation are essential to reduce maternal morbidity and mortality. This is a case report of a 36-year-old primigravida woman at 41 + 6 weeks of gestation with delay in the second stage requiring Neville Barnes forceps delivery. She then went on to have a major postpartum haemorrhage, initially thought to be a result of uterine atony. However, inadequate response to uterotonics led to identification of a cervical buttonhole tear with an intact external os. She required repair under general anaesthesia. A rigid sigmoidoscope was utilised to ensure cervical canal patency during the repair. Previous reports have described annular cervical tears, thought to occur from the extension of a cervical buttonhole tear, but to the best of our knowledge the latter has not previously been reported. The case demonstrates the importance of awareness of such tears and proposes a novel technique for repair with the use of a rigid sigmoidoscope.

## Highlights

• A unique case of cervical buttonhole tear is reported
• Mechanistic continuum of mechanical force to the cervix is suggested; cervical buttonhole tears identified as the first step.
• Suggested surgical technique for a rare tear provided
• Novel use of a sigmoidoscope in cervical tear repair suggested

## 1 Introduction

Postpartum haemorrhage is a significant contributor to maternal morbidity and mortality, with 9% of all maternal deaths between 2016 and 2018 in the United Kingdom and Ireland attributed to bleeding [1]. Trauma, including both genital and cervical, represents the second leading cause of postpartum haemorrhage [2]. Uterotonics are ineffective at managing traumatic postpartum haemorrhage. Interventions that can control traumatic bleeding include vaginal packing and repairing lacerations; if there are unsuccessful, vascular ligation or hysterectomy can be a lifesaving procedure [3].

The majority of genital tract trauma includes perineal tears and episiotomies, alongside obstetric anal sphincter injuries. Cervical tears are an uncommon intrapartum complication with a reported incidence of 0.2% [4]. To date, reported intrapartum cervical tears include annular, bucket handle and avulsion tears, but not buttonhole tears of the cervix [5]. The literature has suggested that complex cervical tears lie on a mechanistic continuum stemming from buttonhole tears, extended to bucket handle and then to annular tears [6]. Nevertheless, there have been no previous reports of buttonhole cervical tears, the first step in this proposed mechanistic process. In view of the rarity of their presentation there is little incentive to have specialised equipment to complete repairs. This paper reports a butthole tear of the anterior cervix following Neville Barnes forceps delivery. It highlights the importance of careful examination of the genital tract after normal and assisted vaginal delivery and reports a novel technique for repair of such tears utilising a rigid sigmoidoscope.

## 2 Case Presentation

A 36-year-old primigravida woman with previous investigation for subfertility presented with an in-vitro fertilisation (IVF) pregnancy. At booking she had a body mass index of 20.55 and was not on regular medication. Apart from iron-deficiency anaemia treated with oral ferrous sulphate, the pregnancy followed an uncomplicated course. Fetal ultrasound growth surveillance demonstrated growth at the 50th centile on a customised growth chart with linear progression. The patient underwent routine induction of labour for being post-dates at 41 + 6 weeks of gestation. Following use of a single dinoprostone pessary (Ferring Pharmaceuticals; Saint Prex, Switzerland) she had a Bishop's score of 7 and underwent artificial rupture of membranes (ARM). ARM was performed on a 5 cm dilated cervix and following siting of an epidural she was commenced on syntocinon. Labour progressed normally, the patient reaching full dilation 6 h after commencing syntocinon. After pushing for 2 h, a lift-out Neville Barnes forceps delivery with a mediolateral episiotomy was performed by an experienced operator. She delivered a 3482-g female infant with Apgar scores at 1 and 5 min of 8 and 9 respectively. She underwent active third-stage labour and the placenta was delivered intact 3 min after delivery of the baby. The episiotomy wound was initially sutured in the room.

Upon completion of third-stage labour, during suturing, the patient had a major postpartum haemorrhage, initially thought to be due uterine atony. She had 1000 ml loss in the delivery room and received a combination of postnatal syntocinon infusion, 250 micrograms of carboprost, 1000 micrograms of misoprostol and 1 g of tranexamic acid. Because there was no improvement with uterotonics, thorough vaginal examination was performed. This revealed a cervical tear. The patient was urgently transferred to the operating theatre due to ongoing bleeding from the genital tract. Examination under anaesthesia was completed for optimal visualisation of the tear. This revealed a patulous cervix with a 4 cm longitudinal buttonhole tear of the anterior cervix at the 12 o'clock position but an intact external cervical os.

The cervical buttonhole tear was repaired with vicryl 2–0 using 4 interrupted sutures spaced 1 cm apart. Prior to repair, concern was raised about controlling suture depth, as friable tissue meant that superficial sutures pulled through the tissue. To ensure canal patency, a sigmoidoscope was inserted – a novel use of that medical equipment. Its solid structure ensured the suture did not catch through as it would with an alternate swab-on-a-stick. After observing to ensure haemostasis, a vaginal pack was inserted at the end of the procedure and removed on postoperative day 1. During the course of events the patient lost a further 200 ml of blood.

After the procedure the patient was transferred to the labour ward for continuous monitoring and one-to-one care. She was commenced on intravenous antibiotics for 24 h. She remained haemodynamically stable postoperatively, with minimal vaginal bleeding, and was transferred to the postnatal ward for routine care. The patient was discharged home on postoperative day 1. She had an uncomplicated postnatal course after discharge, with discharge from regular community midwife follow-up. She was advised to undergo referral to a preterm birth prevention clinic in future pregnancies.

## 3 Discussion

Cervical tears constitute a rare but important cause of postpartum haemorrhage [4]. To date, a variety of tear types have been reported, including annular, bucket handle and avulsion tears [5]. To our knowledge this is the first reported case of a cervical buttonhole tear.

The risk factors for cervical tears remain uncertain, with no consensus. The following have been proposed within the literature as possible risk factors: cervical cerclage, vacuum extraction delivery, nulliparity, early rupture of membranes, precipitous labour and the use of episiotomy [6,7]. Furthermore, prolonged labour is thought to increase the risk of all genital tract trauma, including cervical tears [8]. The presence of cervical fibrotic changes such as those following previous cervical surgery including loop excision, and previous cervical cerclage are also thought to increase risk of intrapartum cervical tears [5]. One case report suggests that external os rigidity on repeated vaginal examination in earlier labour could be a suggestion of possible fibrotic cervix at risk of intrapartum laceration; however, there is no evidence that this is a significant clinical association [11].

The mechanism behind intrapartum cervical tears remains to be elucidated. Several mechanisms have been postulated, including increased strength of contractions, bearing down on a cervix that has not reached full dilatation as well as iatrogenic response to multiple examinations of an oedematous cervix; these, however, remain lacking in conclusive evidence [6]. Previous studies have suggested that complex cervical tears may result from fibrotic changes to the cervix altering the distribution of pressure in an unbalanced fashion, applying more pressure to either the anterior or posterior lip of the cervix [5]. Another suggested mechanism of anterior cervical tears is oedema and necrosis to the cervix as it is compressed between the vertex and the pubic symphysis [8,9]. The suggestion comes from histological findings in individual cases. However, given that there are reported cases of posterior cervical laceration, other pathogenetic mechanisms need to be considered [11]. Alongside these pathogenetic mechanisms, a continuum of mechanical shear forces has been proposed, progressing from a cervical buttonhole tear to a full annular cervical tear [5]. Despite this proposed continuum, there have been no reported cases of a cervical buttonhole to date. This case demonstrates the plausibility of this continuum hypothesis by shedding light on the existence of cervical buttonhole tears. It demonstrates the importance of careful assessment in the presence of unexplained postpartum haemorrhage to identify this potentially hidden form of tear.

No standardised technique of cervical tear repair exists. This is in part due to its rarity. All cervical tears that extend past the internal os and involving the lower uterine segment should have a laparotomy [15]. For small cervical tears less than 2 cm, with minimal bleeding, expectant management can be considered [12,15]. For larger tears or tears resulting in heavy bleeding as in the above case, repair is required. Several important considerations must be factored into the repair. Firstly, there is the likelihood of altering suture tension as postpartum contraction of the uterus occurs and concomitant easement of swelling takes place. This necessitates the use of a suture material that will not cut through the cervix but also is robust enough to withstand the alteration in tension across the repair. In the present case, a braided intermediate-size suture was selected to achieve this. Furthermore, the type of suture technique used needs to be considered, with some suggesting simple interrupted or figure-of-eight interrupted suture technique and others suggesting running continuous, or mattress sutures [8,12]. Literature on skin closure suggests simple interrupted sutures allow drainage and movement; however, they are thought to cause high focal areas of tension at the insertion and exit points of the tissue, unlike mattress or pulley sutures, which aim to dissipate tension [14]. The second consideration is the risk of cervical closure with the suture given its position and access difficulties. Traditionally, prevention of this has been achieved through the use of a swab-on-a-stick. This, however, risks being caught by the suture itself within the compact operating field. The repurposing of pre-existing medical equipment is a well-elucidated approach for rare conditions in which specialised equipment is not available [[16], [17], [18]]. In the report case, a sigmoidoscope was used to ensure cervical patency. Its size and shape predispose it for this use. In addition, its solid form prevents the instrument from being caught within the suture line. It also allows identification of any additional tears within the cervix through its differential colouring. To our knowledge this is the first reported use of a sigmoidoscope for this purpose. Given its beneficial features the use of a sigmoidoscope should be considered to augment repairs of complex cervical tears.

A large retrospective cohort study assessed the effect of cervical lacerations on subsequent birth. It found an increased risk of recurrent cervical laceration (4.9% versus 0.1%, *p* = 0.001) and cervical incompetence (1.9% versus 0.3%, p = 0.001), with older studies suggesting increased spontaneous miscarriage rates [10,13]. There is presently a paucity of evidence on the role of cervical length scanning and use of progesterone in this population. Further research on this is required. As an interim step, referral to a preterm birth prevention clinic and careful outcome auditing are recommended.

## 4 Conclusion

In conclusion, cervical tears are an uncommon intrapartum complication with significant impact on maternal morbidity and mortality. This report presents further evidence that a continuum of complex cervical tears exists. The use of novel surgical techniques, including the use of a sigmoidoscope, should be considered for the repair of these complex tears.
